# Co-infection of human parvovirus B19 with *Plasmodium falciparum* contributes to malaria disease severity in Gabonese patients

**DOI:** 10.1186/1471-2334-13-375

**Published:** 2013-08-15

**Authors:** Nguyen L Toan, Bui T Sy, Le H Song, Hoang V Luong, Nguyen T Binh, Vu Q Binh, Reinhard Kandolf, Thirumalaisamy P Velavan, Peter G Kremsner, C-Thomas Bock

**Affiliations:** 1 Department of Molecular Pathology, Institute of Pathology and Neuropathology, University of Tuebingen, Tuebingen, Germany; 2 Vietnam Military Medical University, Ha Dong, Ha Noi, Viet Nam; 3 Tran Hung Dao Hospital, 108 Institute of Clinical Medical and Pharmaceutical Sciences, Ha Noi, Viet Nam; 4 Vietnam Military Medical Bureau, Ha Noi, Viet Nam; 5 Institute of Tropical Medicine, University of Tübingen, Tübingen, Germany; 6 Centre de Recherche Médicale de Lambaréné (CERMEL), Lambaréné P.B.118, Gabon; 7 Robert Koch Institute, Nordufer 20, D-13353 Berlin, Germany

**Keywords:** Erythrovirus, Human parvovirus B19, *P. falciparum*, Malaria, Co-infection, Gabonese children

## Abstract

**Background:**

High seroprevalence of parvovirus B19 (B19V) coinfection with *Plasmodium falciparum* has been previously reported. However, the impact of B19V-infection on the clinical course of malaria is still elusive. In this study, we investigated the prevalence and clinical significance of B19V co-infection in Gabonese children with malaria.

**Methods:**

B19V prevalence was analyzed in serum samples of 197 Gabonese children with *P. falciparum* malaria and 85 healthy controls using polymerase chain reaction (PCR), enzyme-linked immunosorbent assay (ELISA), and direct DNA-sequencing.

**Results:**

B19V was detected in 29/282 (10.28%) of Gabonese children. B19V was observed more frequently in *P. falciparum* malaria patients (14.21%) in comparison to healthy individuals (1.17%) (*P<*0.001). Notably, the mild-malaria group revealed significantly lower hematocrit levels in B19V/*P. falciparum* co-infection than in *P. falciparum* mono-infection (*P<*0.05). Genetic analysis revealed a predominance of B19V genotype-1 (71.43%) in the studied population. However, B19V-genotype 2 was observed significantly more often in children with severe-malaria than in mild-malaria (*P=*0.04).

**Conclusion:**

Our findings reveal that B19V-infection is frequent in Gabonese children with *P. falciparum* malaria and signifies a possible contribution of B19V on the clinical course of malaria in a genotype-dependent manner. B19V co-infection should be considered as a additional diagnostic measure in malaria patients with life threatening anemia.

## Background

Malaria is one of the major causes of morbidity and mortality in tropical and sub-tropical countries and is caused by the protozoan parasites of the genus *Plasmodium* while *Plasmodium falciparum* (*P. falciparum*) being the most virulent species [1].

*P. falciparum* infected individuals show different clinical phenotypes, which range from asymptomatic infections to severe forms of malaria. The clinical resolution is influenced by multitude of host and parasite factors [2]. Recent studies have underlined the importance of co-infection with human Parvovirus B19 (B19V) in the etiology and pathogenesis of *P. falciparum* malaria both in adults and in children [3-8]. The B19V infection occurs worldwide, with more than 50% are infected with B19V during early childhood with highest reported cases among children from tropical countries [7,9].

B19V belongs to the genus Erythrovirus within the family of *Parvoviridae*[10]. Parvoviruses are non-enveloped DNA viruses that can ably infect mammals [10]. An acute B19V-infection can cause an impaired erythropoiesis [4]. B19V can have a diverse spectrum of clinical manifestations depending on the hematological and the immunological status of the host. Acute B19V-infection can be causative for *erythema infectiosum* (fifth disease), *hydrops fetalis*, and aplastic anemia in individuals with underlying hemolysis [11]. Other manifestations include arthritis [12,13], myocarditis [14,15], vasculitic syndromes [16], neurological disorders [17], and hepatic inflammation [18,19].

B19V-infection has been demonstrated to elevate severe anemia caused by *P. falciparum* among young children in the Republic of Nigeria [7]. The B19V-infection outbreak in Nigeria reported that 54% of children with *P. falciparum* associated severe anemia (hematocrit level, <20%) showed an evidence of B19V-infection [7]. Similar studies from Ghana, Papua New Guinea, and Kenya support the finding that B19V can play an important role in the etiology of severe anemia in children living in malaria endemic areas [8,20,21]. However, other studies from Malawi [22] and Kenya [23] observed little evidence of acute B19V-infection in severe anemia in children with malaria.

In the present case–control study we utilized 282 healthy and *P. falciparum* infected Gabonese children from sub-Sahara Africa. We aim to investigate the prevalence of B19V-infection from this malaria endemic area and further sought to determine the relationship of B19V and *P. falciparum* co-infection on the etiopathogenesis of *P. falciparum* malaria.

## Methods

### Study subjects

282 children were recruited at the Albert Schweitzer Hospital, Lambaréné, Gabon, and the Centre Hospitalier de Libreville, Libreville, Gabon. The investigated cohort is from a matched pair, case–control study, to compare severe and mild malaria in Gabon. Details of the study cohort are as described elsewhere [24,25]. Serum samples of the patients were cryo-freezed and stored as different aliquots at −80°C until use. 197/282 individuals were infected with *P. falciparum* with well-characterized clinical profiles (Table 1)*.* 85/282 children served as healthy controls and had no evidence and/or clinical signs of *P. falciparum* infection during recruitment [26]. The healthy control individuals were chosen of the same sex, age, and locality and the exclusion criteria were asymptomatic *P. falciparum* infection and indications for concurrent diseases and malnutrition. Among the 197 *P. falciparum* infected children, individuals were further classified in two sub-groups either as severe (n=97) or mild malaria (n=100) based on clinical signs, symptoms and parasite load with clinical profiles as shown in Table 1. Clinical presentation of the severity of *P. falciparum* infection has been described previously [25,27-29].

**Table 1:** **Characteristics of Gabonese children with *P. falciparum *malaria according clinical presentation**

**Characteristics**	**Mild malaria (n=97)**	**Severe malaria (n=100)**	***P***
Age (Month)	44.90	44.23	n.s.
Male/female	35/62	39/61	n.s.
Systolic blood pressure (mmHg)	103	93	<0.01
Heart frequency (beats/min)	120	130	<0.001
Respiratory rate (breaths/min)	35	41	<0.001
Hematocrit (Proportion of 1.0)	3290.6	2143.5	<0.0001
Hemoglobin (g/L)	>80	<50	<0.0001
Lactate (mmol/L)	2.4	2.9	<0.05
Parasite × 1000/μL	26.492	407.08	<0.0001
Schizontaemia (n)	0	24	<0.001

n.s. = not significant, values are given as median, values for male/female are given as numbers.

Severe malaria was defined as severe anemia (hemoglobin <50 g/L) and/or hyperparasitemia (> 250,000 parasites/ml corresponding to >10% infected erythrocytes) and facultative signs of severe malaria, such as cerebral malaria, convulsions, hypoglycemia, and respiratory distress.

Individuals with mild malaria were chosen from patients of the same sex, age, and locality, and admitted as soon as the severe cases were enrolled for this study. For mild malaria, the following criteria were considered: parasitemia between 1,000/l and 50,000/l on admission, no schizonts in the periphery, malarial pigment containing circulating leukocytes <50/μl, hemoglobin >80 g/L, platelets >50/nl, leukocytes <12/nl, and blood glucose >50 mg/dl (Table 1). Exclusion criteria were signs of severe malaria and/or other acute infections and prior hospitalization for any reasons to exclude possible severe malaria in the history and intake of antimalarial drugs within the preceding weeks.

### Polymerase chain reaction (PCR)

Nucleic acid was extracted from patient sera using High Pure Viral Nucleic Acid Kit (Roche, Grenzach-Wyhlen, Germany) according to the manufacturer’s instruction. The detection of B19V-DNA was tested by nested polymerase chain reaction (nPCR) as described previously using primer pairs specific for the VP1/VP2-coding regions [30]. All samples tested positive for B19V were reconfirmed with an independent second round of PCR which amplified the NS1/VP1u-region [31]. PCR conditions employed were described as in previous studies [31]. In order to prevent assay contamination, sample processing (DNA-extraction, template preparation, centrifugation, aliquoting, and master mix preparation) and PCR-amplification were performed in dedicated laboratory facilities certified for molecular diagnostics and especially for PCR, using standard precautions. Specificity of PCR products were reconfirmed by DNA-sequencing. The B19V-DNA sequences were aligned with published B19V-genome sequences [GenBank accession number: genotype 1A: M13178, genotype 1B: DQ357064, genotype 2: AY064476 and AY044266; and genotype 3: AX003421, AY083234].

### Quantitative real-time PCR for B19V-DNA

The B19V-DNA load was determined in the serum by B19V-specific quantitative real-time PCR (qPCR) using the GeneAmp 7300 sequence analyser (TaqMan Sequence Detection System; Applied Biosystems Perkin-Elmer, Foster City, CA) The primers, probes and program conditions were used as described previously [30,32]. The specificity and sensitivity of the B19V-qPCR was determined using the WHO international standards for B19V-DNA (NIBSC Code 99/800) containing 10^5^ IU/ml B19V DNA along with integrated B19V-negative control plasmids. In order to standardize the qPCR, increasing dilutions of B19V plasmid DNA (6.5 × 10^4^ to 6.5 × 10^1^ B19V IU/ml) were included. The lower sensitivity of the B19V-qPCR assay was found to be 2 × 10^2^ IU/ml. Samples were analyzed in duplicates including extraction of nucleic acids.

### DNA sequence analysis

DNA fragments spanning the coding NS1/VP1u region from nt 2355–2690 (nt. position according to AF162273) were amplified by nPCR as described previously for all B19V-DNA positive samples [19,31]. The sequencing reaction was performed with 5 μl of purified PCR products, 2 μl BigDye Terminator Cycle Sequencing reaction (Life Technologies Corp., USA), and 15 pmol primer pairs. The PCR products were sequenced in sense and antisense direction using primers as described previously [31]. The primers used for sequencing were sense (n-P5F) 5′-CGTGAACTGTTAGTTGGGGTTGA-3′ and antisense (n-P5R) 5′-AATTGCTGATACACAGCTTTAG-3′. The DNA sequences were analyzed using ABI Prism Genetic Analyser 3100C (Applied Biosystems, Foster City, USA).

### B19V serology evaluation

Anti-B19V-IgM and anti-B19V-IgG (VP1/VP2) were analyzed by enzyme-linked immunosorbent assay (ELISA) (Parvovirus B19-IgM; Parvovirus B19-IgG, DxSelect™ FocusDiagnostics, Germany) according to the manufacturer’s instructions. An Index Value >1.20 measured of the serum samples in the ELISA assay indicated the presence of B19V IgG or IgM antibodies. The anti-B19V IgM ELISA assay has a reported sensitivity of 89% and specificity of 98% and the anti-B19V IgG ELISA assay a sensitivity of 96% and specificity of 97%, respectively.

### B19V-genotype analysis

B19V-sequences were aligned using CLUSTALW (http://www.ebi.ac.uk/Tools/msa/clustalw2/) and GENEDOC2.5 (http://www.psc.edu/biomed/genedoc). Genetic distances were calculated using the Kimura two-parameter model incorporated into the MEGA5.0 [33]. Phylogenetic trees were reconstructed by MEGA5.0 using the Neighbour-Joining method [34]. The robustness of inferred relationships was assessed with bootstrapping based on 1000 replicate data sets (bootstrap value < 40 were not shown on the tree).

B19V-sequences available from the GenBank database were used as reference sequences for sequence alignment [GenBank accession numbers: genotype 1A: AB030694, AF113323, AF162273, and M13178; genotype 1B: DQ357064, DQ357065, and KF417555; genotype 2: AY064476 and AY044266; and genotype 3: AX003421, AY083234]. The GenBank accession numbers for the nucleotide sequences of B19V-samples determined in this study are from KF309501 to KF309528.

### Statistical analysis

Statistical analysis was performed using Statview 4.57 (http://www.statview.com),JMP Statistical Discovery Software, version 5.0.1 (http://www.jmp.com), and Prism5 (version 5.01, GraphPad Software, San Diego California USA, http://www.graphpad.com). A *P-*value <0.05 was considered to be statistically significant in all statistical computations. We computed Fisher’s exact test and the Mann–Whitney *U* test and Spearman’s Rho test for our analysis as accordingly.

### Ethical approval

The study was approved by the ethics committee of the International Foundation for the Albert Schweitzer Hospital in Lambaréné, Gabon. Informed written consent for participation in the study was obtained from a parent or guardian of the children.

## Results

### Prevalence of B19V-DNA and anti-B19V-antibodies

**Figure 1:**
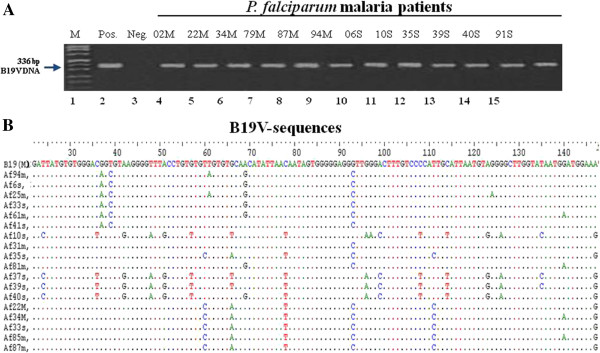
**Qualitative assessment of B19V genomes in Gabonese children with *P. falciparum*. (A)** Representative samples of the qualitative detection of B19V-DNA in serum of *P. falciparum* malaria patients using nPCR (lanes 4 to 15). DNA size marker, positive control, and negative control are shown in lane 1, 2 and 3, respectively. **(B)** B19V sequences of B19V-positive patients were aligned with reference B19V sequences using CLUSTALW (http://www.ebi.ac.uk/Tools/msa/clustalw2/), BioEdit (http://www.mbio.ncsu.edu/BioEdit), and GENEDOC_2.5 (http://www.psc.edu/biomed/genedoc) [GenBank accession No. genotype 1A: M13178, genotype 1B: DQ357064, genotype 2: AY064476 and AY044266; and genotype 3: AX003421, AY083234]. Sequence homologies are denoted as dots and nucleotide exchanges are shown in letters. The ruler at top of the sequences is numbered according to the B19V-NS1/VP1 sequences (nt 1 correspond to nt 2355).

In order to determine the prevalence of B19V-DNA in sera of the *P. falciparum* malaria patients we performed nPCR amplifying the B19V VP1/VP2 and B19V NS1/VP1u region. Representative nPCR results using B19V-NS1/VP1u specific primers generating a 336bp B19V-NS1/VP1 fragment are shown in Figure 1A. NS1/VP1u amplicons were confirmed by DNA-sequencing (Figure 1B). Sequencing analysis revealed that sequences differ between the B19V isolates, thereby excluding cross-contamination of patient samples representing patient-specific B19V isolates.

**Figure 2:**
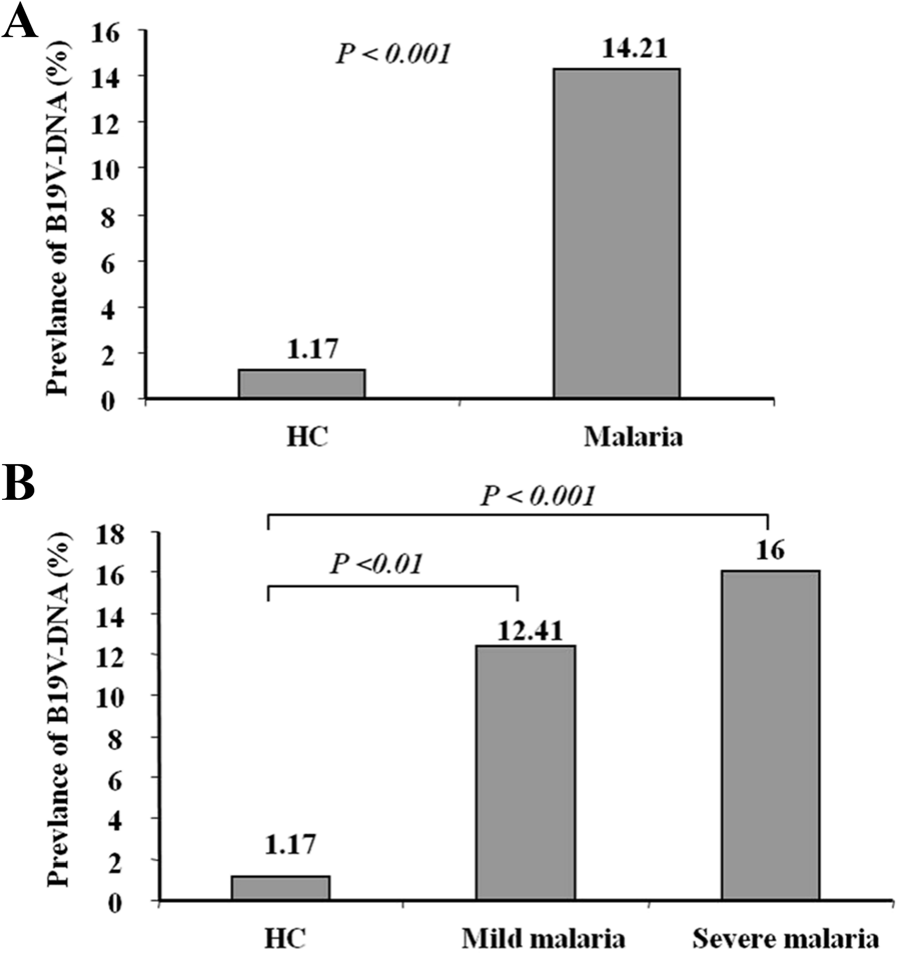
**Prevalence of B19V genomes in children with *P. falciparum *and healthy controls. (A)** Prevalence of B19V-genome detection in Gabonese patients with *P. falciparum* malaria and healthy control group (HC) (odds ratio (OR) = 12.08 (CI_95_ [1.62-90.28], two tailed Fisher’s exact test, *P*<0.001). **(B)** Distribution of B19V genomes in Gabonese patients with *P. falciparum* malaria according to their clinical presentation of malaria (mild and severe) and in healthy control group (HC) (OR =10.52 (CI_95_ [1.34 - 82.6] and OR=13.6 (CI_95_ [1.77 - 104.7], respectively, two tailed Fisher’s exact test, *P*<0.01).

Investigation on the prevalence of B19V in patients with mild, severe *P. falciparum* malaria and in controls showed that B19V-DNA were detectable in 29/282 (10.28%) of Gabonese children. The prevalence of B19V-DNA was significantly higher in patients with *P. falciparum* malaria compared to healthy subjects (14.21% vs. 1.17%, *P<*0.001; Figure 2A). However, the proportion of B19V-DNA detection was significantly higher in the severe and mild malaria patient groups in comparison to the healthy subjects following the profile: healthy control < mild malaria < severe malaria (1.17%, 12.37%, and 16%, respectively; Figure 2B) (*P*<0.01 and *P<*0.001; respectively).

Twenty-eight B19V-DNA positive samples of patients with *P. falciparum* were examined for anti-B19V-IgM and anti-B19V-IgG. In total, B19V-IgM and B19V-IgG antibodies were detected in 20/28 (71.43%) and 19/28 (67.86%) serum samples, respectively. Overlapping anti-B19V-IgM and anti-B19V-IgG were detected in 11/28 (39.28%) cases, whereas 9/28 (32.14%) patients were anti-B19V-IgM-positive and anti-B19V-IgG-negative and 8/28 (28.57%) of patients were anti-B19V-IgG-positive and anti-B19V-IgM-negative.

Additionally simultaneous detection of B19V-DNA and B19V-IgM antibodies in 71,4% (20/28) of the serum samples is indicative for an acute or recently acute B19V-infection rather than a persistence of B19V-DNA.

### B19V-loads, parasitemia of P. falciparum, and anemia in patients with P. falciparum malaria

**Figure 3:**
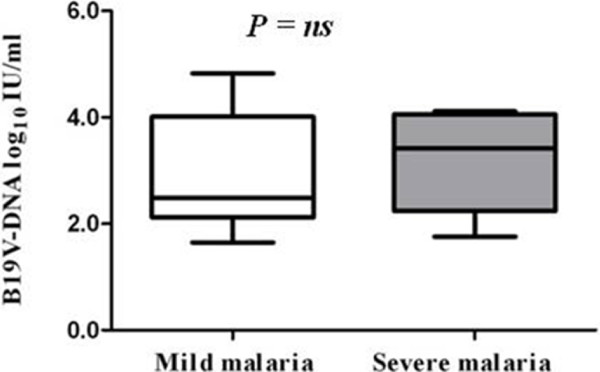
**Serum B19V-DNA loads in co-infected B19V*/P. falciparum/ *patients according to clinical presentation of mild and severe malaria.** Serum B19V-DNA loads showed no significant differences between the mild and severe malaria patient groups (median, 2.48 log10 IU/ml (range 1.65-4.83 IU/ml) vs 3.42 log10 IU/ml (range 1.76-4.11 IU/ml), *P*=ns (Mann–Whitney *U* test).

In order to examine the B19V replication levels of B19V/*P. falciparum* co-infections, we examined B19V loads in the serum of the Gabonese children with malaria. The B19V loads in sera of B19V*/P. falciparum* co-infected patients were 3.08 log10 (range 1.65-4.83) IU/ml. No significant differences of the B19V loads of sera of mild malaria patients (median, 2.48 log10 IU/ml [range 1.65-4.83 IU/ml]) compared to severe malaria patients (median, 3.42 log10 IU/ml [range 1.76-4.11 IU/ml]) were observed, *P>*0.05 (Figure 3).

Additionally, the parasitemia of *Plasmodium spp.* did not differ significantly in B19V/*P. falciparum* co-infected patients compared to mono-infections of *P. falciparum* (201.910 ± 184.018 vs. 201.570 ± 276.973 parasites/μL, respectively, *P>*0.05; Table 1). However, in the mild malaria group the parasitemia of *P. falciparum* patients co-infected with B19V was marginally higher (41.900 ± 38.818 parasites/μL) compared to *P. falciparum* mono-infection (23.600 ± 31.277 parasites/μL; *P*=0.06).

**Figure 4:**
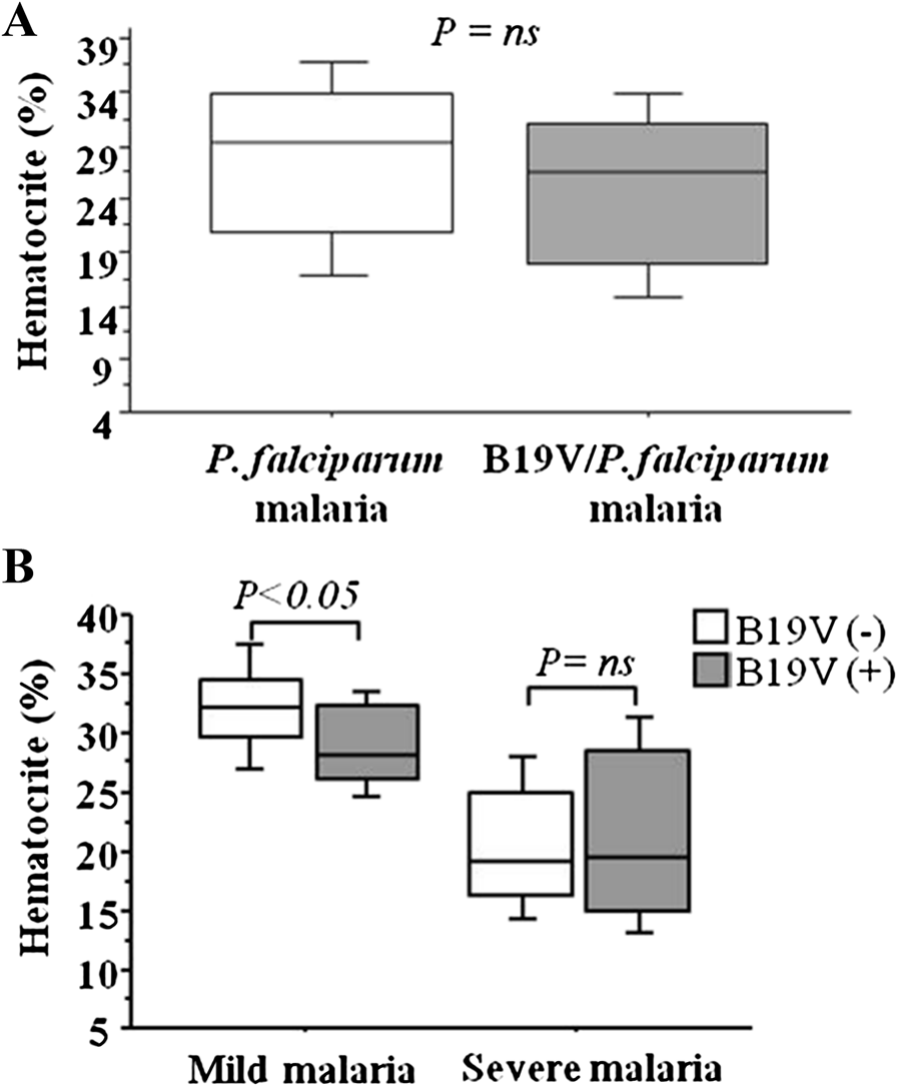
**Hematocrit levels of co-infected B19V/*P. falciparum *and mono-infected *P. falciparum *patients. (A)** Hematocrit levels of mono-infected *P. falciparum* (white box) and B19V*/P. falciparum* co-infected (grey box) are denoted. **(B)** Hematocrit levels of mild and severe malaria patients are shown. In mild malaria patients, the hematocrit level co-infected with *B19V/P. falciparum* patients was significantly lower in comparison to mono-infected *P. falciparum* patients. *P*<0.05 values are statistically significant (Mann–Whitney *U* test).

The investigation of hematocrit levels in mild malaria patients revealed that hematocrit levels were significantly lower in patients with B19V/*P. falciparum* co-infection in comparison to *P. falciparum* mono-infection (*P*<0.05; Figure 4A). However, in severe malaria patients hematocrit levels were not altered when compared to B19V/*P. falciparum* coinfection with *P. falciparum* mono-infection (*P*>0.05; Figure 4B).

### Distribution of B19V-genotypes in Gabonese children

Currently, three Erythrovirus genotypes (genotype 1, 2 and 3) have been described [35,36] whereas B19V-genotype 1 seems to be the predominant genotype worldwide in recent years [37]. In order to examine the distribution of B19V-genotypes in Gabonese children with *P. falciparum* malaria we analysed B19V-positive samples of the patient cohort using molecular epidemiological methods. Sequencing analysis of the sub-genomic B19V NS1/VP1u region indicated that both B19V genotypes 1 and 2 were detectable in Gabonese children. However, B19V-genotype 1 was observed to be more frequent than genotype 2 (20 of 28 (71.43%) vs. 8 of 28 (28.57%), whilst genotype 3 could not be detected in the study population.

**Figure 5:**
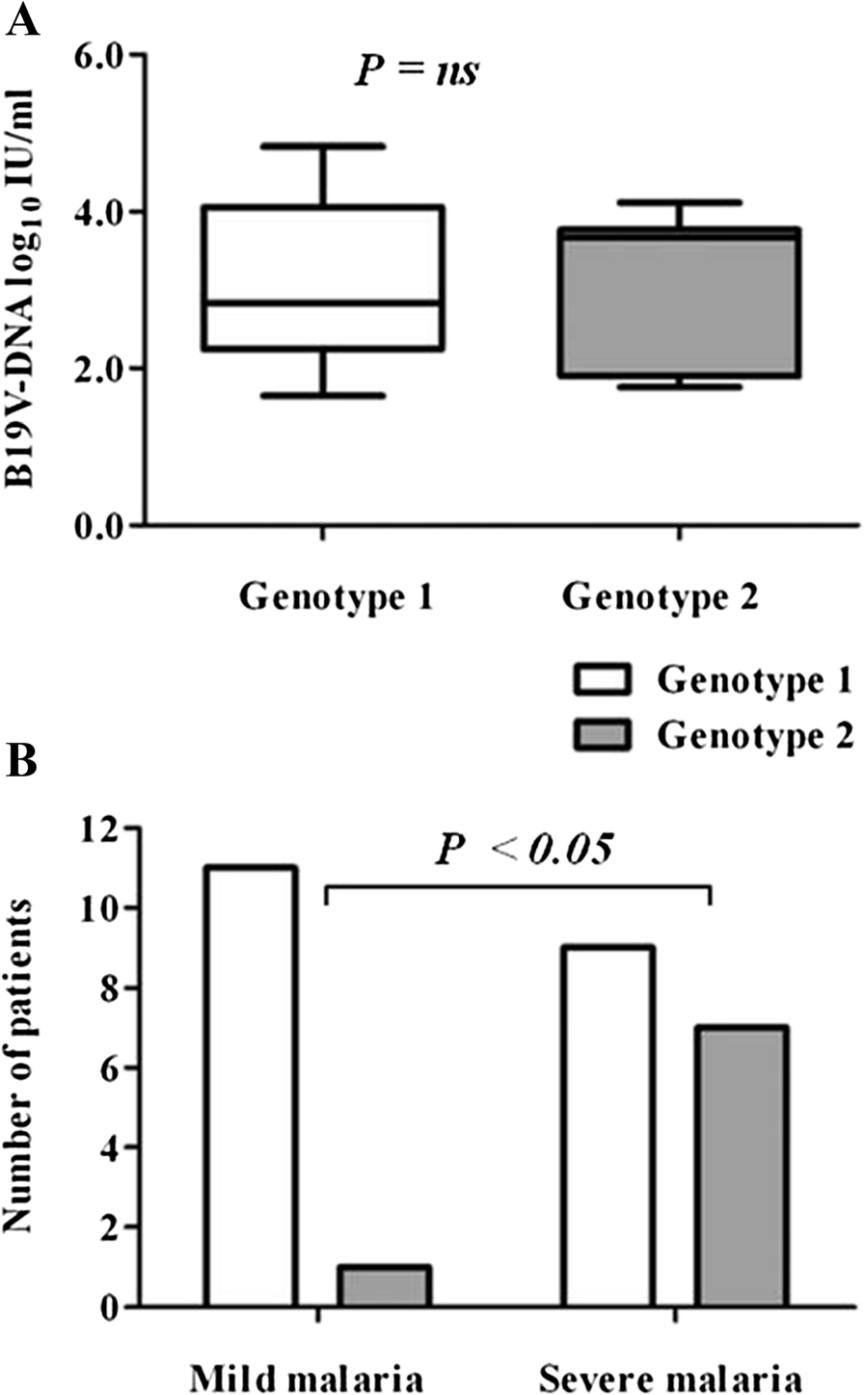
**B19V genotype-specific distribution and viral loads in B19V/*P. falciparum *coinfected malaria patients. (A)** B19V genotype-specific viral loads was not significantly different between the malaria patient groups (median, 2.83 log10 IU/ml (range 1.65-4.83 IU/ml) vs 3.67 log10 IU/ml (range 1.76-4.11 IU/ml), *P*=ns (Mann–Whitney *U* test). **(B)** Prevalence of B19V genotypes 1 and 2 in mild and severe malaria patient groups. The prevalence of B19V-genotype 2 was significantly higher in severe malaria than in mild malaria (OR = 8.56 (CI_95_ [0.88-83.1], one-tailed Fisher’s exact test, *P*<0.05).

To determine the contribution of B19V-genotypes on the course of *P. falciparum* malaria (mild and severe), we examined the distribution of B19V-genotypes in serum of *P. falciparum* malaria patients. No significant differences in the levels of parasitemia, hemoglobin and hematocrit in the serum of the malaria patients across the different B19V genotypes were observed (data not shown). Additionally, B19V-DNA loads showed no significant differences (*P*>0.05; Figure 5A). However, B19V-genotype 2 was more significantly detected in patients with severe malaria (7/16) compared to patients with mild malaria (1/12) (Figure 5B; *P*<0.05; one tailed Fisher’s exact test).

**Figure 6:**
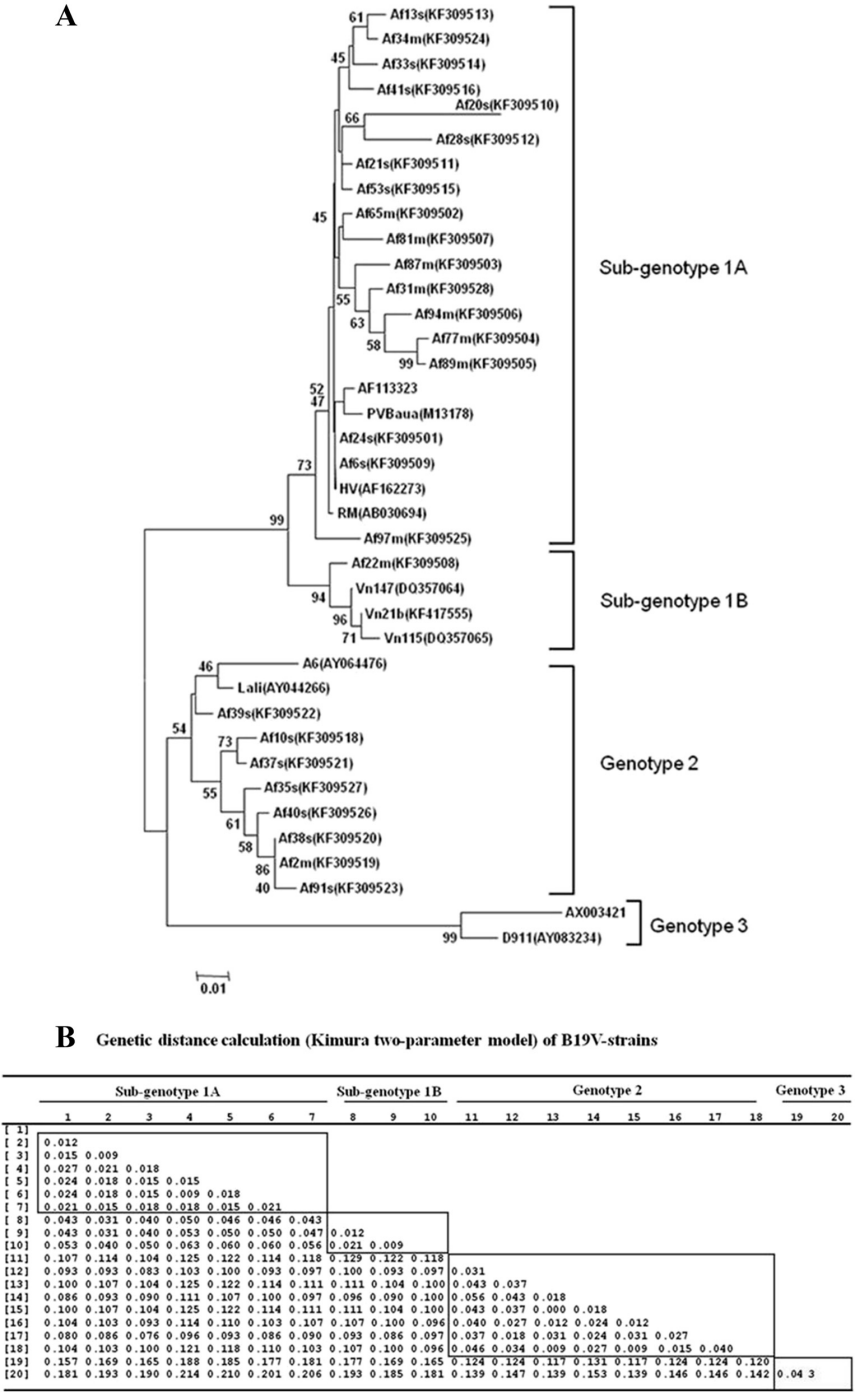
**Phylogenetic analysis of the B19V-NS1/VP1u region. (A)** The phylogenetic tree has been inferred from the B19V-NS1/VP1u region (nt 2355 to nt 2690) of 39 B19V-samples. Topology was constructed by MEGA 5.0 software using the Neighbor-joining method with bootstrap values (in percent) indicating numbers from 1,000 replicates tested (values <40 were not shown). Major lineages of B19V are shown as branches. The two major B19V-subtypes 1A and 1B are shown [31]. Scale bar at bottom indicates the number of nucleotide substitutions per site. Patient codes and the corresponding GenBank accession number are denoted (e.g., Af31m(KF309528)). Reference B19V-sequences were GenBank accession No. AB030694, AF113323, M13178 and AF162273 for B19V-genotype 1A, DQ357064, DQ357065, and KF417555 for B19V-genotype 1B, AY064476 and AY044266 for B19V-genotype 2, and AX003421 and AY083234 for B19V-genotype 3. **(B)** Genetic distance calculation (Kimura two-parameter model) of B19V-strains. Numbers at the left denote patient-specific B19V-strains and B19V-reference strains [Genbank numbers are denoted]: [1] M131178; [2] AB030694; [3] Af21s (KF309511); [4] Af13s (KF309513); [5] Af33s (KF309514); [6] Af34m; [7] Af41s (KF309516); [8] Af22m (KF309508); [9] DQ357064; [10] DQ357065; [11] AY064476; [12] AY044266; [13] Af2m (KF309519); [14] Af37s (KF3095021); [15] Af38s (KF3095020); [16] Af35s (KF3095027); [17] Af39s (KF3095022); [18] Af40s (KF3095026); [19] AY083234; [20] AX003421. B19V-reference strains are genotype 1: [1], [2], genotype 2: [11], [12], and genotype 3: [19], [20]. Boxes represent data of the genetic distance calculation of the same genotype and sub-genotype.

Recently, our studies demonstrated that B19V-genotype 1 can be classified into at least two B19V sub-genotypes 1, B19V-1A and B19V-1B [31]. In order to analyze the B19V sub-genotype in Gabonese children the sub-genomic NS1/VP1u region of the B19V-positive patient samples were compared to B19V reference sequences from the database using phylogenetic analysis. Nineteen B19V-sequences from the studied Gabonese clustered in a single clade of B19V-genotype 1A (67.85%) whereas one B19V isolate clustered with the other clade of B19V-subgenotype 1B (Af22m [GenBank: KF309508]). Eight B19V-sequences clustered with the genotype 2 reference group (28.57%; Figure 6A).

The genetic distances among the newly detected B19V- and reference strains were calculated based on the Kimura two-parameter model using MEGA5.0 software. The results of the genetic distance analysis of the sub-genomic B19V NS1/VP1u region of the B19V samples are shown in Figure 6B. The genetic distances among the B19V-strains within each B19V-genotype were very low, ranging from 0.009 to 0.063 for B19V-genotype 1 (1A and 1B)(Figure 6B, No. 1–10) and from 0.000 to 0.056 for B19V-genotype 2 (Figure 6B, No 11–18) while B19V-genotype 3 was not detected in our study. Further analysis showed that the genetic distances determined in B19V sub-genotypes 1A and 1B were also low with highest value of 0.063 (Figure 6B, No 4/genotype 1A and No 10/genotype 1B). In contrast, the mean distance between different B19V-genotypes was higher than the mean intra-genotypic divergence (Figure 6B, >0.129). Overall, the average genetic distance was 0.086 among all B19V-genotypes. The genotypic identification of B19V-strains was completely accurate (100%) using both the diagnostic nucleotide and distance-based/phylogenetic approaches.

## Discussion

Malaria due to *P. falciparum* infection still pose major health problem in developing countries and in many sub-Saharan countries such as Gabon. In regions with high endemicity for *P. falciparum* infection, the majority of children with malaria present a mild form of the disease. A small proportion of individuals infected with *P. falciparum* develop severe forms of malaria with a higher risk of mortality and morbidity [38]. The association of B19V with aplastic crisis in hemolytic diseases such as sickle cell anemia is well reported, however, their role in coinfection with *P. falciparum* malaria is yet controversial. Anemia in malaria is multifactorial and mainly due to haemolysis and dyserythropoiesis [39]. Recently, two studies in children of Papua New Guinea showed that certain common infections, and especially B19V-infection can play a critical role in the etiology of severe anemia besides other factors, such as malnutrition and iron deficiency, in a highly endemic area for malaria [21,40]. This finding was further supported by yet another study showing that high B19V IgM levels were significantly associated with severe anemia in Kenyan children [20]. B19V-infection can result in severe anemia since B19V selectively inhibits and lysates actively replicating erythroid progenitor cells [11,41]. Notably, target cells of B19V are erythroid cells and erythroid precursors which are shared by *P. falciparum* probably resulting in case of co-infection in more severe anemia [7,21,42-44].

A previous study has shown a high prevalence of B19V and *P. falciparum* co-infection in Nigeria [7]. In contrast, other studies from Malawi [22], Ghana [8], and Kenya [20,45] found little evidence of acute B19V infection in children (either anemic or control).

In accordance to the study of Nigeria, we could demonstrate that B19V genomes were detectable in 10.28% of young Gabonese individuals while the frequency of B19V was significantly higher in *P. falciparum* malaria patients than in healthy children (*P*<0.001, Figure 2). Children with severe *P. falciparum* malaria showed the highest rate of B19V infection (16.0%) when compared to mild malaria (12.37%). These findings suggest that B19V may probably play a role in the pathogenesis of *P. falciparum* malaria following a B19V prevalence profile of healthy control < mild malaria < severe malaria. In accordance with our findings on increased B19V detection in *P. falciparum* malaria patients, a previous report suggested that depression of cell-mediated immunity in *P. falciparum* infection might favour co-infection with opportunistic pathogens including B19V [46].

With regard to clinical outcome of *P. falciparum* malaria previous studies have shown a failure of recovery of hemoglobin after effective anti-malarial treatment and delayed recovery of *P. falciparum* malaria after subsidence of B19V infection [3,42]. Furthermore, the association of B19V infection with hemoglobin levels may be related to individual variability. Studies have shown previously that a significant decrease in hemoglobin levels can occur in patients with *P. falciparum* malaria [3,47]. These findings corroborate well with our observation of significantly lower hematocrit levels in B19V/*P. falciparum* co-infected patients in comparison to *P. falciparum* mono-infection (*P*<0.05; Figure 4).

Evaluation of B19V loads, which showed mainly acute B19V-infection due to positive B19V-IgM results (67,86%), and parasitemia showed no significant differences between mild and severe malaria groups in the studied population possibly postulate that there is no direct or indirect interaction of B19V and *P. falciparum* with regards to viral replication. However, a slightly but not significant increase in parasitemia could be observed in mild malaria co-infected with B19V in comparison to mono-infection of *P. falciparum* (*P*>0.05; Table 1).

Different genotypes of viruses, such as human immunodeficiency virus (HIV) and hepatitis B and C viruses, show different susceptibility to disease progression or response to antiviral treatment. We therefore analysed the distribution of B19V-genotypes in the studied *P. falciparum* patients. Only *Erythrovirus* (B19V)-genotypes 1 and 2 were observed in the studied population, however genotype 3 cannot be observed in our study population. B19V-genotype 1, and especially B19V-genotype 1A, was the most prevalent genotype in Gabonese children. This is in accordance with previous studies showing a predominance of B19V-genotype 1 worldwide [31,35,48,49]. Interestingly, B19V genotype 2 was found in 28.6% of the young Gabonese children. These findings were unexpected as recent reports indicated that B19V-genotype 1 and 2 follow an age-dependent distribution while B19V genotype 1 is detectable mainly in younger people (<45 years) and genotype 2 can mostly be observed in patients older than 60 years [15,50]. However, a geographical distribution of B19V genotype 2 could not be completely excluded as it has been shown for genotype 3 [51].

We did not observe any significant differences in parasitemia, hemoglobin and hematocrit levels of *P. falciparum* malaria patients across the different B19V genotypes 1 and 2. However, genotype 2 was detected significantly more often in children with severe-malaria compared to children with mild-malaria (P<0.05). The detected association of B19V-genotype 2 with severe malaria needs further analysis, although, one can postulate that variability in the host immune response to distinct B19V-genotypes may correspond with the severity of anemia in B19V/*P. falciparum* patients.

## Conclusions

Overall to our knowledge this is the first study which supports the hypothesis that an acute B19V infection can modulate the clinical course of *P. falciparum* malaria in Gabonese children. Additionally, our results point towards increased B19V co-infection among young children with *P. falciparum* malaria. Two of the three Erythrovirus genotypes are detected in these populations while genotype 2 remained more frequent in children with severe malaria. The hematocrit levels of B19V/*P. falciparum* co-infected mild-malaria patients were significantly decreased as a consequence of co-infection. These findings indicate that the clinical outcome of *P. falciparum* malaria in Gabonese children is influenced by B19V co-infection and should be considered as a diagnostic measure in patients with worsening anemia and persistent fever in spite of effective anti-malarial therapy. However the precise molecular mechanisms involved in B19V co-infection in the context of pathogenesis of *P. falciparum* malaria yet needs to be understood and warrants further analysis.

## Abbreviations

B19V: Parvovirus B19; PCR: Polymerase chain reaction; nPCR: nested PCR; qPCR: quantitative real time PCR; ELISA: Enzyme-linked immunosorbent assay.

## Competing interests

The authors declare that they have no competing interests.

## Authors’ contributions

PGK, RK, NLT, and CTB conceived the study and designed the research; HVL, NTB, VQB, and TPV participated in developing study instruments; NLT, BTS. LHS, and TPV conducted the research; NLT, BTS, and TPV performed statistical analysis; PGK, RK, NLT, LHS, BTS, TPV and CTB participated in writing and helped to draft the manuscript. All authors read and approved the final manuscript.

## Pre-publication history

The pre-publication history for this paper can be accessed here:

http://www.biomedcentral.com/1471-2334/13/375/prepub
